# The First Fossil Representatives of the Sawfly Genera *Emphytus* and *Empria* from the upper Miocene of France (Hymenoptera, Tenthredinidae) †

**DOI:** 10.3390/insects13020218

**Published:** 2022-02-21

**Authors:** André Nel, Rose Marie Sammut, Meicai Wei, Gengyun Niu, Corentin Jouault

**Affiliations:** 1 Institut de Systématique, Évolution, Biodiversité (ISYEB), Muséum National d’Histoire Naturelle, CNRS, Sorbonne Université, EPHE, Université des Antilles, CP50, 57 rue Cuvier, 75005 Paris, France; jouaultc0@gmail.com; 2 20 Allée du Sal, 15130 Arpajon sur Cère, France; couscous.sam@wanadoo.fr; 3 College of Life Sciences, Jiangxi Normal University, Nanchang 330022, China; weimc@126.com (M.W.); gyniu@126.com (G.N.); 4 Univ. Rennes, CNRS, Géosciences Rennes, UMR 6118, 35000 Rennes, France; 5 CNRS, UMR 5554 Institut des Sciences de l’Évolution de Montpellier, Place Eugène Bataillon, 34095 Montpellier, France

**Keywords:** Insecta, Symphyta, Allantinae, Late Miocene, new species

## Abstract

**Simple Summary:**

Two sawflies belonging to the tenthredinid genera *Emphytus* and *Empria* are described from upper Miocene diatomite paleolakes from Southern France. They are compared with all their extant and fossil relative genera. The previously described fossil representatives of these two genera are discussed. These fossils are of great interest for dating in future phylogenetic analyses of the Tenthredinidae.

**Abstract:**

*Emphytusmiocenicus* sp. nov., first fossil representative of this genus, is described from the upper Miocene of the diatom paleolake of Montagne d’Andance (Ardèche, France). Its placement is ensured by an in-depth comparison with all the extant and fossil genera of the subfamily Allantinae. The representatives of *Emphytus* are distributed in the Palearctic, Nearctic, and Oriental regions. *Empria sammuti* sp. nov., second representative of the latter genus, is described from the latest Miocene of the diatom paleolake of Sainte-Reine (Cantal, France). The placement of this new species is based on a detailed comparison with the extant genera of the tribe Empriini. The larvae of the extant *Emphytus* and *Empria* spp. are known to be phytophagous on angiosperm leaves of several families, all present as fossils in the taphocenoses of la Montagne d’Andance and Sainte-Reine. *Emphytus miocenicus* sp. nov. represents the oldest record of this genus and of its crown group, corroborating the estimate of a middle Eocene–middle Oligocene age for its stem group. Throughout our study, it appears that the first described fossil of the genus *Empria*, *E. oligocaenica,* from the Oligocene of Germany, needs to be revised and redescribed. It should preferably be treated until the revision as *incertae sedis* in Allantinae *sensu lato*.

## 1. Introduction

The family Tenthredinidae is the most speciose extant sawfly family [1]. Nevertheless, its fossil record remains scarce, especially for the Cretaceous, even if recent phylogenetic analyses have suggested a Cretaceous age for the diversification of the family into its modern subfamilies, especially for the Allantinae [2,3]. Described fossil Allantinae comprise *Athalia vetuecclesiae* Wappler et al., 2005, *Athalia* (?) *wheeleri* (Cockerell, 1906), *Empria oligocaenica* (Meunier, 1923), *E. bruesi* Rohwer, 1908, *Eriocampa disjecta* Cockerell, 1922, *Er. Pristina* Cockerell, 1910, *Er. Scudderi* Brues, 1908, *Er. Synthetica* Cockerell, 1911, *Er. Tulameenensis* Rice, 1968, *Er. Wheeleri* Cockerell, 1906, *Hylotomites robusta* Meunier, 1914, *Palaeathalia laiyangensis* Zhang, 1985, *Palaeotaxonus trivittatus* Rohwer, 1908, *Pa. typicus* Brues, 1908, *Pa. vetus* Cockerell, 1917, *Pseudosiobla campbelli* Rice, 1968, *Ps. Megoura* Cockerell, 1907, *Ps. Misera* (Brues, 1908), *Taxonus nortoni* Scudder, 1890, and *T. vetustus* (Heer, 1849) [1]. All these fossils are from Cenozoic era (mainly from the Oligocene) except for the early Cretaceous *Palaeathalia laiyangensis*. Vilhelmsen and Engel [4] (p. 272) indicated that “The fossil record of Tenthredinoidea is substantial; putative stem group taxa are known from the Late Jurassic and Early Cretaceous, whereas fossils that can be placed in extant families do not occur until the Tertiary”. Thus, there is a significant discrepancy between the time divergence estimates and the fossil record of tenthredinid subfamilies. Based on recent analyses and correlation with the Angiosperms diversification [1], it was proposed that the Tenthredinidae have diversified rapidly, paralleling the diversification of flowering plants. To further study this hypothesis, a better documentation of their fossil record is needed. In the near future, study of the Cretaceous or the Paleocene periods will be crucial to decipher the evolution of Tenthredinidae.

Here, we describe the first fossil representative of the genus *Emphytus*, from the upper Miocene, and the second fossil species of the genus *Empria,* from the latest Miocene, both from French deposits.

## 2. Material and Methods

The holotype of *Emphytus miocenicus* sp. nov. originates from the Miocene Konservat-Laggerstätte of the Montagne d’Andance (Saint-Bauzile, Ardèche, France). It was found *ca.* 35 years ago. The diatomitic unit from which the specimen originated was deposited in a lake formed by a maar crater [5] under a warm and humid climate [6], with possibly seasonal character. This lake was probably calm and shallow [7], surrounded by an immense heterogeneous forest with an undergrowth of ferns [8,9]. Over time, this lake was filled with diatomite, forming a rock strata up to 30 m thick in some localities [10]. This diatomite is a soft siliceous rock in which was preserved the insects that drowned in the lakes at the time. These stratigraphic layers of diatomite represent a reducing environment that has allowed a remarkable conservation of organic matter that is mummified. The age of this deposit corresponds to the Tortonian–Messinian boundary, *ca.* 7.30 ± 0.15 Ma [11].

The holotype of *Empria sammuti* sp. nov. originates from the Miocene Konservat-Laggerstätte of Sainte-Reine (Fourfouilloux, Virargues village), near Murat (Cantal, France). The diatomitic unit from which the specimen originated was deposited in a lake probably formed by a maar crater, under a rather high altitude (*ca.* 1000 m). The age of this deposit corresponds to the latest Miocene, *ca*. 5.60 ± 0.3 Ma [12]. After Roiron [13], the flora indicates an important cooling for the period.

The specimens were studied using a stereomicroscope Nikon SMZ25 and the photographs were taken with a Nikon D800 mounted on the previously mentioned stereomicroscope or using a Canon 50D with an attached Canon 65 MPE camera lens and mounted on an automated stacking rail (StackShot). The photographs were treated with graphic software. All images are digitally stacked photomicrographic composites of several individual focal planes, which were obtained using Helicon Focus 6.7. The figures were composed with Adobe Illustrator CC2019 and Photoshop CC2019.

We follow the tenthredinid classification proposed by Wei and Nie [14] rather than Taeger et al. [1] for the treatment of the genus *Emphytus*. Niu et al. [2] recently separated a subfamily Megabelesinae from the Allantinae on the basis of a molecular phylogenetic analysis. We follow the wing venation nomenclature of Goulet and Huber [15].

## 3. Results

Systematic paleontology.
Order Hymenoptera Linnaeus, 1758.
Family Tenthredinidae Latreille, 1818.
Subfamily Allantinae Rohwer, 1911.
Tribe Allantini Rohwer 1911.
Genus *Emphytus* Klug, 1815.

*Emphytus miocenicus* sp. nov.

**Figure 1:**
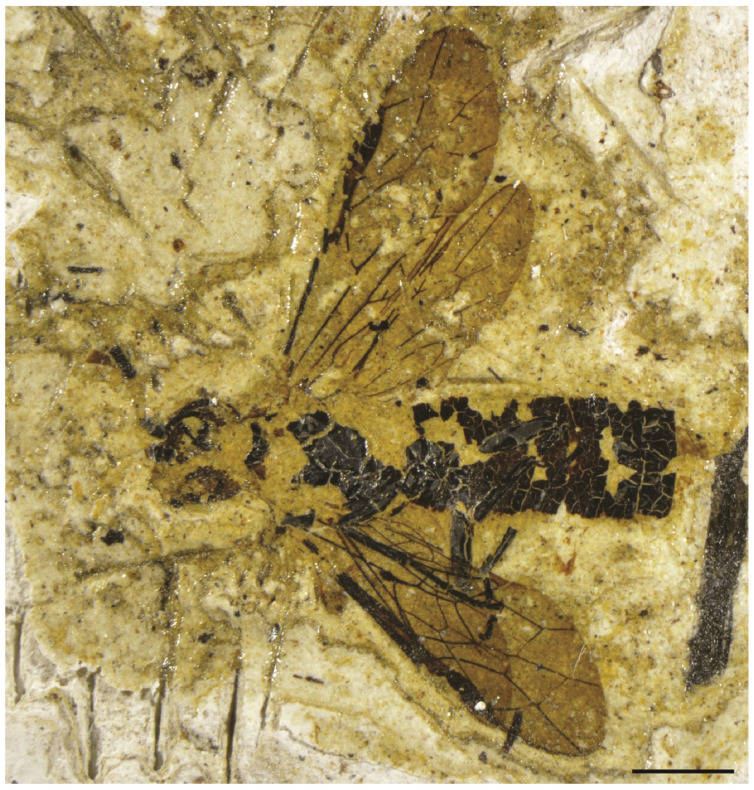
*Emphytus miocenicus* sp. nov., holotype IGR-PAL-2859. Photograph of general habitus. Scale bar = 2 mm.

**Figure 2:**
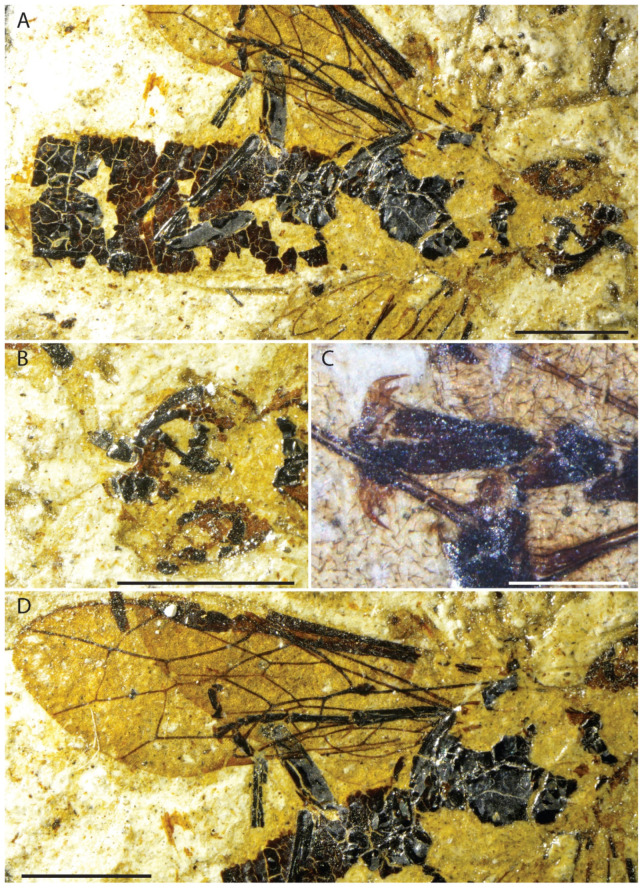
*Emphytus miocenicus* sp. nov., holotype IGR-PAL-2859. Photographs. (**A**) Body. (**B**) Head. (**C**) Tarsal claw. (**D**) Left fore and hind wings. Scale bars = 2 mm.

**Figure 3:**
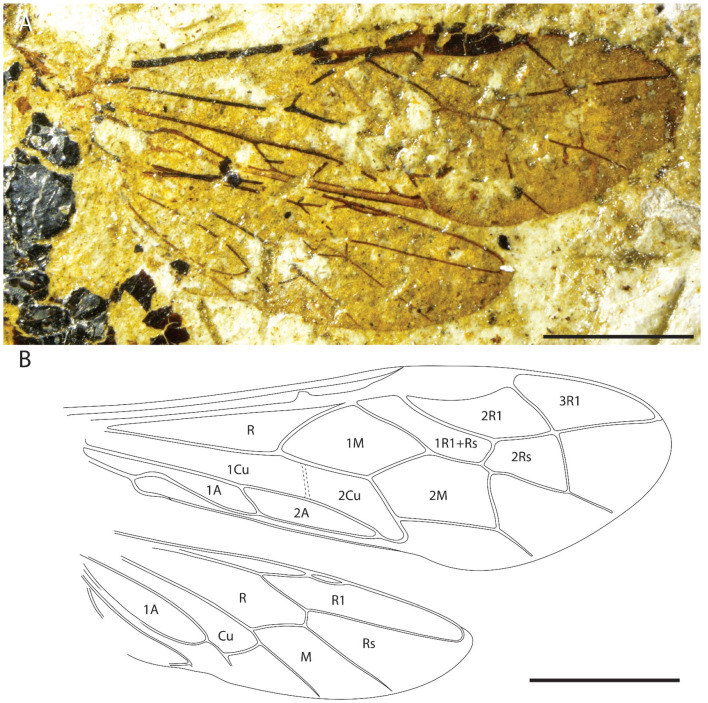
*Emphytus miocenicus* sp. nov., holotype IGR-PAL-2859. Right fore and hind wings. (**A**) Photograph. (**B**) Reconstructions with wing venation and cell names labeled. Scale bars = 2 mm.

Figure 1, Figure 2 and Figure 3.

urn:lsid:zoobank.org:act:1697F94A-5445-4E15-8D7F-13D4C6835E71

Etymology. Named after the Miocene period.

Material. Holotype specimen IGR-PAL-2859 (a compression of a nearly complete adult, but with head poorly preserved), housed in the Geological Department and Museum of the University of Rennes, France (IGR).

Diagnosis. A rather large species (compared to the extant European species), with body length *ca.* 11.0 mm; wings uniformly infuscate; pterostigma unicolored, dark brown; abdomen uniformly black; all femora, tibiae, and tarsi uniformly black; forewing vein 3A reaching posterior wing margin.

Description. Body length *ca.* 11.0 mm.

Color. As the fossil is a compression mummy, the differences in the color pattern have some significance because they reflect the original differences in coloration. Body black with faint traces of brown on the abdomen; compound eyes brown; legs black; wings infuscate with apical third slightly darker than basal two-thirds.

Head poorly preserved, *ca.* 2.0 mm long, 2.0 mm wide; compound eyes 0.8 mm apart; mouthparts not preserved; seven antennomeres preserved but antennae not complete; third antennomere as long as fourth. Thorax *ca.* 3.3 mm long, 2.4 mm wide, mainly smooth and shining, covered dorsally with very small punctuations. Abdomen shining without surface sculpture, 2.4 mm wide, but apex missing. Mid legs with femur 1.5 mm long, tibia 1.55 mm long, tarsus 2.0 mm long, longer than tibia; inner tibial spur 0.3 mm long; claw with an inner tooth 0.2 mm long, apical tooth 0.4 mm long, basal lobe small but distinct, rounded; hind legs with femur 1.9 mm long, tibia 2.7 mm long, tarsi not preserved. Forewing 7.7 mm long, 2.7 mm wide; pterostigma 1.7 mm long, 0.4 mm wide; no first free sector of Rs between the cells 1R1 and Rs; cell 1R1 + Rs 1.9 mm long; cell 2Rs 1.2 mm long; nervulus (1cu-a) situated just beside middle of cell 1M; vein a oblique and elongate; vein 3A reaching posterior wing margin. Hind wing 5.6 mm long, 1.7 mm wide; cells Rs and M opened; apex of cell 1A 0.4 mm beside apex of cell Cu; vein 3A very faint but reaching posterior wing margin.

Taxonomic remarks

Following the key to tenthredinid subfamilies of Goulet [16], the new fossil falls in the Allantinae because of the following characters: “fore and hind wings as long as or longer than length of abdomen”; “veins lm-cu and Cu, of forewing meeting at an angle of 140°”; “forewing with main axis of vein M and that of 1m-cu subparallel”; “vein R of forewing not deviated between junctions of M and Sc”; “vein 2A and 3A of forewing complete and markedly constricted”; “crossvein of anal cell of forewing developed and long”; “vein Rs+M of forewing near junction with R straight”. Following Haris’s [17] key to tenthredinid subfamilies, the Nematinae and the Heterarthrinae are excluded because of the presence of the basal part of vein 2r-rs in forewing. Affinities with the Susaninae are also excluded because of the very different shape of the anal veins and base of Rs at point of fusion of M with R [18]. The Dolerinae are excluded because the new fossil has a vein 2r-m separating the cell 1R1 + Rs (vein Rs missing) from cell 2Rs. The subcosta straight, without widened area in front of pterostigma, excludes the Tenthredininae. The Selandriinae are excluded because of the presence of an anal crossvein a, and the base of Rs+M near Sc+R straight [19]. The Blennocampinae are excluded because the anal cell of the forewing is complete, without stem, and the crossvein of the anal cell is elongate in the new fossil. Lacourt [20] proposed a concept of the Blennocampinae that comprises several genera listed in the Allantinae by Taeger et al. [1]. Thus, it appears that there is no real consensus on the classification of the Tenthredinidae and that new phylogenomic analyses will help to clarify the systematics of the family.

The forewing 2A + 3A separated from 1A by a distinct anal vein, the veins M and lm-cu subparallel, and the forewing veins lm-cu and Cu1 forming an angle of 140°, present on the specimen studied here, are characteristic of the Allantinae present in the new fossil and corroborate the keys used to tentatively place this fossil.

Within the Allantinae, affinities with the Eriocampini (*Eriocampa* Hartig, 1837, *Pseudosiobla* Ashmead, 1898, *Dimorphopteryx* Ashmead, 1898, *Armitarsus* Malaise, 1931, *Corymbas* Konow, 1903, etc.) are excluded because of the veins M and Rs+M meeting Sc+R at the same point, plus the mesoscutellum and the mesonotum without deep punctures in the new fossil, and the affinities with the Acidiophorini (*Acidiophora* Konow, 1899) are excluded because the pterostigma is rather broad and the tarsal claws are bifid [21,22]. The Adamasini (genus *Dinax* Konow, 1897 = *Adamas* Malaise, 1945) are excluded because of their simple claw, the short crossvein a, and the presence of the first free sector of Rs in forewing [23,24].

The main differences between the Empriini and Allantini concern the clypeus, the mandibles, and the thorax, which are not or poorly preserved in the new fossil. Thus, we proposed an in-depth comparison with all the genera encompassed by these two tribes.

*Blennallantus* Wei, 1998, *Cladiucha* Konow, 1902, *Acladiucha* Wei, 1997, and *Ateloza* Enderlein, 1920 have the forewing vein 2A + 3A atrophied at apex so that the basal anal cell is open [25,26,27]. The presence vs. absence of the “forewing first free sector of Rs between the cells 1R1 and Rs” is variable in *Cladiucha* [28]. *Acladiucha* has also a hind wing cell M closed.

The two characteristics “forewing first free sector of Rs missing between 1r-rs and M+Rs” and “vein 3A relatively developed reaching posterior wing margin” exclude affinities with *Indostegia* Malaise, 1934 [29]. *Haymatus* Smith, 1979 has a hind wing cell M and a forewing first free sector of Rs present, unlike the new fossil [30]. *Somanica* Smith, 1979, *Megabeleses* Takeuchi, 1952, *Tripidobeleses* Wei, 1997a, *Taxoblenus* Wei & Nie, 1999a, *Clypea* Malaise, 1961, *Ferna* Malaise, 1961, *Emphystegia* Malaise, 1961, *Hemibeleses* Takeuchi, 1929, *Belesempria* Wei & Nie, 1997, *Dasmithius* Xiao, 1987, *Eriocampopsis* Takeuchi, 1952, *Allanempria* Wei, 1998, *Isotaxonus* Saini & Vasu, 2001, *Anisotaxonus* Saini & Vasu, 1998, *Ungulia* Malaise, 1961, *Kambaitia* Malaise, 1961, *Kambaitina* Malaise, 1961, *Tala* Malaise, 1935, *Heptapotamius* Malaise, 1935, *Indotaxonus* Malaise, 1957, *Neacidiophora* Enslin, 1911, *Netroceros* Konow, 1896, *Paranetroceros* Koch, 1998, *Mucheana* Koch, 1998, *Pseudoneacidiophora* Koch, 1998, *Hainandaonia* Wei & Nie, 1998, *Gulingia* Wei et al., 1997, *Metallotala* Wei, 1997, *Thecatiphyta* Wei in Blank et al., 2009 (new name for *Sainia* Wei, 1997), *Ilithyiana* Wei, Nie & Taeger, 2006 (=*Ilithyia* Wei, 1997), *Emphytopsis* Wei & Nie, 1998, and *Abeleses* Enslin, 1911 also have a forewing first free sector of Rs [25,26,31,32,33,34,35,36,37,38,39,40,41,42,43,44,45,46,47,48,49,50,51,52,53,54,55,56]. *Darjilingia* Malaise, 1934 also has a forewing with first free sector of Rs and the subapical tooth of the claw much longer than the apical, unlike the new fossil [57,58].

The absence of the hind wing cell M in the new fossil excludes the genera *Triallan* Smith, 2014, *Sunoxa* Cameron, 1899, and *Eusunoxa* Enslin, 1911 [59,60,61]. *Apethymorpha* Wei, 1997 has the hind wing cell Rs closed. The third antennomere is as long as the fourth and the absence of the forewing first free sector of Rs exclude affinities with the genera *Phrontosoma* MacGillivray, 1908, *Gulingia* Wei et al., 1997, and *Allantunicus* Smith in Smith & Schiefer, 1997. *Phrontosoma* and *Gulingia* have also a hind wing cell M [43]. *Monostegia* Costa, 185, *Monsoma* MacGillivray, 1908, *Formosempria* Takeuchi, 1929, and *Kattakumia* Zhelokhovtsev, 1964 (=*Mongolempria* Ermolenko, 1968) have also a forewing first free sector of Rs [62,63,64,65]. *Monostegia* and *Monsoma* further have a hind wing cell M [30,66], preventing attribution of the new specimen to these genera. *Harpiphorus* Hartig, 1837 has the forewing cell M with a distinct dorsal petiole and the cell M of hind wing closed [24]. *Conobeleses* Wei, 1997a has also a forewing cell M with a distinct dorsal petiole [25]. The genera *Athalia* Leach, 1817 and *Hypsathalia* Benson, 1962 generally have also such petiole, a forewing first free sector of Rs, and hind wing cell Rs and M [67]. *Hennedyella* Forsius, 1935 and *Hennedyia* Cameron, 1891 have a small inner tooth of the tarsal claws, unlike the new fossil [67]. *Ametastegia* Costa, 1882 (including *Ocla* Malaise, 1957) differs from the new fossil because of the vein a clearly less oblique, and a very long third antennomere [30]. The presence *vs.* absence of the forewing first free sector of Rs is variable in this genus. *Ocla* has the hind wing without closed cell M, but its nervulus (1cu-a) is curved and the basal lobe of claw is more pronounced than in the new fossil [32].

*Eopsis* Benson, 1959 has also a forewing first free sector of Rs, *plus* a very broad pterostigma [68]. *Emphytopsis* Wei & Nie, 1998 has also a forewing first free sector of Rs, *plus* the teeth of the claws of nearly the same lengths [48,69]. *Taxonus* Hartig, 1837 (with *Allomorpha* Cameron, 1876, *Parasiobla* Ashmead, 1898, *Nesotaxonus* Rohwer, 1910) has a forewing first free sector of Rs, and shorter hind wing anal veins [30,70]. *Aphilodyctium* Ashmead, 1898 and *Probleta* Konow, 1908 (=*Protoprobleta* Malaise, 1949) share with the new fossil the hind wing cells Rs and M opened, but they have a forewing first free sector of Rs, and a rudimentary vein 3A [19,30,71]. *Antholcus* Konow, 1904 has a hind wing cell M and an acute basal lobe of the tarsal claws [19]. *Shenia* Wei in Wei & Nie, 2005 and *Thaumatotaxonus* Wei in Wei & Nie, 1999 have a forewing first free sector of Rs and the cells Rs and M closed [72,73].

The species of the genus *Empria* Lepeletier, 1828 have the forewing first free sector of Rs absent or present and the hind wing cell M opened or closed, tarsal claws with or without an inner tooth, but they have paired opalescent white spots on abdominal terga 2 to 5 or more [30], while the new fossil has none.

All the genera of the tribe Xenapateini sensu Koch [74], except *Takeuchiella* Malaise, 1935, viz. *Allantidea* Rohwer, 1912, *Nepala* Muche, 1986, *Neoxenapates* Forsius, 1934, *Xenapatidea* Malaise, 1957, and *Xenapates* Kirby, 1882, share with the new fossil the forewing vein 3A reaching the posterior wing margin, but all differ from it in the presence of the forewing first free sector of Rs, and the hind wing cells Rs and M closed [74,75,76,77,78]. Some species of *Takeuchiella* have no closed hind wing cells Rs and M, but they still have a forewing first free sector of Rs. *Togashia* Wei, 1997 differs from the new fossil in the presence of a forewing first free sector of Rs and a large basal lobe of the claw [79,80]. *Jinia* Wei & Nie, 1999 also has a forewing first free sector of Rs and a closed hind wing cell M [80].

*Caiina* Wei, 2004 has the two teeth of its claws of the same lengths and a closed hind wing cell M [81], while their length is different and the cell M open in the new fossil. *Neotaxonus* Saini & Vasu, 1996 has a closed hind wing cell M and a pronounced basal lobe of the claws [82]. *Monostegidea* Rohwer, 1915 has a closed hind wing cell M and no basal lobe of claw [83].

The two genera *Tritobrachia* Enderlein, 1920 and *Nagamasaia* Togashi, 1988 of the subtribe Nagamacina have claws without basal lobe, unlike the new fossil. *Tritobrachia* has no forewing first free sector of Rs, while *Nagamasaia* has one [72,84]. *Empronus* Malaise, 1935 has also claws without basal lobe, plus a closed hind wing cell M [85]. *Mallachiella* Malaise, 1934 has also a very minute basal lobe of claw [86]. *Hemkuntus* Saini & Deep, 1992 has no basal lobe of claw [87]. *Hemocla* Wei, 1995 shares with the new fossil the absence of the forewing first free sector of Rs, and the opened hind wing cell M, but it also has no basal lobe of the claw [88].

*Macremphytus* MacGillivray, 1908, *Asiemphytus* Malaise, 1947, *Parabeleses* Wei & Nie, 1998, *Filixungulia* Wei, 1997, and *Plumalaminia* Wei, 1997 share with the new fossil the absence of the forewing first free sector of Rs, but they have a closed hind wing cell M [30,47,55,89,90,91,92]. *Apethymus* Benson, 1939 also shares with the new fossil the absence of the forewing first free sector of Rs, but it has the teeth of its tarsal claws nearly of the same lengths [93,94].

The two genera *Athlophorus* Burmeister, 1847 and *Hemathlophorus* Malaise, 1945 of the subtribe Athlophorina *sensu* Wei & Nie [49] (=tribe Athlophorini sensu Lacourt [22]) have no forewing first free sector of Rs, and hind wing without closed cell M. *Athlophorus* differs from the new fossil in the forewing veins M and Rs + M not meeting Sc + R at the same point [94,95,96]. *Hemathlophorus* differs from the new fossil in a venation similar to that of *Athlophorus* and the subapical tooth of the claw longer and stronger than the apical one [88,97]. *Stenempria* Wei, 1997 and *Fernophytus* Wei, 1997 have also no forewing first free sector of Rs and hind wing without closed cell M, but the former differs from the new fossil in the claw without inner tooth and basal lobe, and the later in the claw without basal lobe and inner tooth very small and remote from apical tooth [90,98].

*Paralinomorpha* Koch, 1988a has no forewing first free sector of Rs, but differs from the new fossil in the very large basal lobe of the claw and the hind wing cell subclosed [99]. *Yushengliua* Wei & Nie, 1999b also has a larger basal lobe of the claw than in the new fossil [71,100]. *Nervobeleses* Wei et al., 2006 (new name for *Doleroides* Nie & Wei, 1998) also has no forewing first free sector of Rs but differs from the new fossil in the two teeth of the claw of the same lengths and a strong basal lobe [101].

*Allantus* Panzer, 1801, *Emphytus* Klug, 1815, *Taxonemphytus* Malaise, 1947, *Mimathlophorus* Malaise, 1947, *Linomorpha* Malaise, 1947, *Apethymus* Benson, 1939 (junior syn. *Kjellia* Malaise, 1947, after Koch [102]), *Maghrebiella* Lacourt, 1989, and *Parallantus* Wei & Nie, 1998d share with the new fossil the elongate vein 3A, the absence of the forewing first free sector of Rs (but Will [103] has shown that some specimens of *Emphytus* can have this vein), the hind wing cells Rs and M opened, and the basal lobe of the claw small and rounded [104]. *Allantus* differs from the new fossil in the nervulus 1cu-a of the forewing close to the base of vein M [71,104]. *Emphytus* Klug, 1815 has this vein in a more distal position, meeting the cell 1M at basal third, as in the new fossil. Both *Allantus* and *Emphytus* have acute basal lobes of the claw, unlike *Maghrebiella*. The shape of the claw in the new fossil strongly resembles that in *Emphytus* and strongly differs from those of *Allantus* and *Maghrebiella* [104]. *Taxonemphytus* and *Mimathlophorus* have an inner tooth stronger and longer than the apical one, unlike the new fossil [88]; also, the apex of the hind wing cell 2A is touching that of cell 1A in these two genera, unlike in the new fossil. *Linomorpha* has a very particular cell 2Rs with a very short crossvein 2r-m.

*Apethymus* and *Hemiphytus* Malaise, 1947 have claws and a venation very similar to that of the new fossil, but the forewing vein 3A is absent in *Apethymus*, and the length of cell 1R1+Rs is twice as long as cell 2Rs in *Hemiphytus*, while it is only 1.75 times as long as cell 2Rs in the new fossil [88,105]. *Taxonemphytus* has the inner tooth of claw longer than apical one [88]. *Parallantus* has the two teeth of the claws of the same length and the forewing anal cell without crossvein, unlike the new fossil [49].

*Stenemphytus* Wei & Nie, 1999 has no forewing first free sector of Rs, the hind wing cells Rs and M opened, but it differs from the new fossil in the cell1R1 + Rs two and a half times as long as the cell 2Rs, while they are only 1.75 times as long as cell 2Rs in the new fossil [79].

The latest Oligocene genus *Hylotomites* Meunier, 1914 was originally placed near the argid genus *Hylotoma*, but Taeger et al. [1] placed it in the Allantinae without giving arguments. Therefore, as far as we know, this genus has never been revised. It differs from the new fossil in the forewing cell M with a distinct dorsal petiole (“cellule radiale distinctement appendiculée”) [106]. The fossil genus *Palaeotaxonus* Brues, 1908 (lowermost Oligocene of Florissant Fm., Colorado, USA) has a forewing first free sector of Rs [107]. *Palaeathalia* Zhang, 1985 (Early Cretaceous, Laiyang Fm., Liaoning, China) was originally considered as a Tenthredinidae [108], placed in the Allantinae by Abe and Smith [109] and Taeger et al. [1], but considered as a Tenthredinoidea by Ronquist et al. [110]. *Palaeathalia* also differs from the new fossil in the presence of a forewing first free sector of Rs [108].

The new fossil shares with *Emphytus* a majority of its preserved characters to the exclusion of the other allantine genera. Taeger et al. [1] considered that *Emphytus* is a subgenus of *Allantus*, but Lacourt [104] separated the two taxa *Allantus* and *Emphytus*, followed by Magis [111], Pesarini [112], and Wei and Nie [14]. Here, we follow this treatment and propose to include it in the genus *Emphytus*.

Tribe Empriini Rohwer 1911.
Genus *Empria* Lepeletier 1828.

*Empria sammuti* sp. nov.

**Figure 4:**
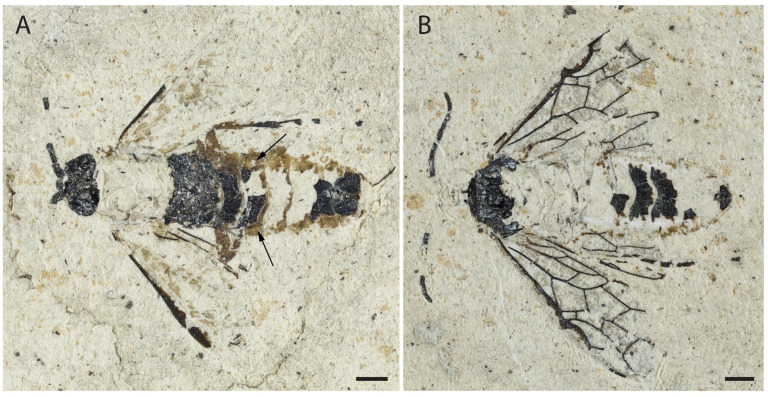
*Empria sammuti* sp. nov., holotype IGR-PAL-2860. Photographs of habitus. (**A**) Imprint, arrows show less-sclerotized zones. (**B**) Counterimprint. Scale bars = 1 mm.

**Figure 5:**
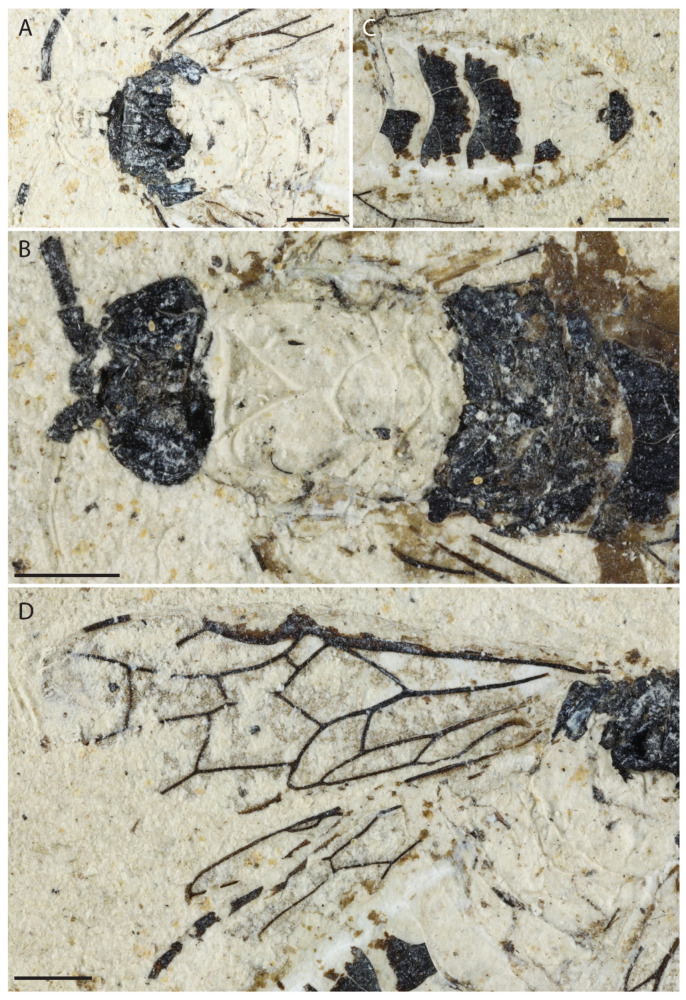
*Empria sammuti* sp. nov., holotype IGR-PAL-2860. Photographs. (**A**) Head and mesosoma. (**B**) Metasoma. (**C**) Head and mesosoma. (**D**) Wing venation. Scale bars = 1 mm.

Figure 4 and Figure 5.

urn:lsid:zoobank.org:act:24A47217-78D2-4137-8EC5-0D3A2C5AF241

Etymology. Named after Claude Sammut, who allowed us to study the type specimen and donated to the University of Rennes. It is to be treated as a noun in the genitive case.

Material. Holotype specimen IGR-PAL-2860 (a compression of a nearly complete adult), housed in the Geological Department and Museum of the University of Rennes, France (IGR).

Diagnosis. Body black except brown lateral parts of abdominal tergites; wing infuscate with costal margin black; hind femur brown, hind tibia with basal half brown and apical one black, hind tarsus black; anterior margin of clypeus has two weak lateral bumps separated by a very weak concavity; forewing first free sector of Rs between cells 1R1 and 1Rs present.

Description. Body length 10.2 mm.

Color. As for above, the differences in the color of the different parts of the body reflect the original differences because it is a compression mummy. Body black with lateral traces of brown on abdomen; compound eyes black; legs brown with hind tarsus darker than femur and tibia; wings infuscate with costal margin darker.

Head well preserved, 1.3 mm long, 1.8 mm wide; compound eyes large, 0.5 mm apart; labrum 0.1 mm long, covering the mandibles; anterior margin of clypeus has two weak lateral bumps separated by a very weak concavity; antenna 3.2 mm long, with nine antennomeres preserved, third as long as fourth, longer than more distal ones. Thorax 4.0 mm long, 2.5 mm wide, mainly smooth and shining, covered dorsally with very small punctuations; medial length of metapostnotum is 0.5 of its maximal length. Abdomen shining without surface sculpture, 5.4 mm long, 2.7 mm wide; median part of tergites sclerotized and black, lateral sides brown and less sclerotized; short ovipositor present but poorly preserved. Only hind legs visible, femur 1.4 mm long, tibia 1.4 mm long, tarsus 2.7 mm long, longer than tibia; claws not preserved. Forewing 7.5 mm long, 2.8 mm wide; pterostigma 1.4 mm long, 0.5 mm wide; first free sector of Rs between cells 1R1 and 1Rs present; cell 1R1 0.5 mm long; cell 1Rs 1.0 mm long; cell 2Rs 1.3 mm long; nervulus (1cu-a) situated at middle of cell 1M; vein a oblique and elongate. Hind wing 5.0 mm long; cells Rs and M opened; cells 1A and Cu not visible.

Taxonomic remarks

Following the key to tenthredinid subfamilies of Goulet [16], the new fossil falls in the Allantinae because of the following characteristics: “fore and hind wings as long as or longer than length of abdomen”; “veins lm-cu and Cu, of forewing meeting at an angle of 120°–150°”; “forewing with main axis of vein M and that of 1m-cu subparallel”; “vein R of forewing not deviated between junctions of M and Sc”; “vein 2A and 3A of forewing complete and markedly constricted”; “crossvein of anal cell of forewing developed and long”; “vein Rs + M of forewing near junction with R straight”. After this key, the only character that precludes affinities with the Allantinae and rather suggests affinities with the Selandriinae is “veins lm-cu and Cu, of forewing meeting at an angle of 120°”, while it would be between 140° and 150° in Allantinae. However, this character is not stable in the Allantinae (*ca.* 120° in *Dinax* Konow, 1897) [29]. The Selandriinae are excluded because of the presence of an anal crossvein a, and the base of Rs + M near Sc + R straight [19].

Within the Allantinae, affinities with the Eriocampini are excluded because of the veins M and Rs + M meeting Sc + R at the same point, plus the mesoscutellum and the mesonotum without deep punctures in the new fossil. Affinities with the Acidiophorini (*Acidiophora* Konow, 1899) are because the pterostigma is rather broad. The Adamasini are excluded because of their short crossvein a [23,113]. After Goulet [16], the Allantini are characterized by “medial length of postnotum of metathorax 0.8 or more of maximal length of postnotum, about halfway between side and midline” and “anterior margin of clypeus widely concave” vs. “medial length of postnotum of metathorax 0.7 or less of maximal length of postnotum, halfway between side and midline” and “anterior margin of clypeus less widely concave than above” in the Empriini. In the new fossil, the medial length of metapostnotum is 0.5 of its maximal length, and the anterior margin of clypeus has two weak lateral bumps separated by a very weak concavity, in accordance with an attribution to the Empriini.

Within this tribe, affinities with the genera *Monosoma* MacGillivray 1908, *Monostegia* Costa 1859, *Phrontosoma* MacGillivray 1908, *Plumalaminia* Wei, 1997, *Harpiphorus* Hartig, 1837, and *Haymatus* Smith, 1979 are excluded because the hind wing cell M is opened in the new fossil [24,30,90]. *Stenempria* Wei, 1997 has cells 1R1 and 1Rs fused [90]. *Blennallantus* Wei, 1998 and *Allanempria* Wei, 1998 have a flat clypeus as in the new fossil, but a metapostnotum flat, medially not constricted, unlike the new fossil [26]. *Dasmithius* Xiao, 1987 has a very shallowly and broadly circularly emarginated clypeus, and cell 2Rs as long as cell 1Rs, unlike the new fossil [35]. *Ungulia* Malaise, 1961 has the forewing cell 2Rs as long as cell 1Rs and the anal vein nearly perpendicular; *Oralia* Malaise, 1961 (with junior synonym *Himindica* Saini, 1996) has also a nearly perpendicular anal vein, and the fourth antennomere as long as fifth, forewing vein 3A relatively developed reaching posterior wing margin, and the hind wing vein 3A well developed and elongate [33].

*Somanica* Smith, 1979 and *Aphilodyctium* Ashmead 1898 have a more pronounced concavity of the clypeus than in the new fossil [30]. *Protemphytus* Rohwer 1909 has the forewing cells 1R1 and 1Rs combined [114]. *Hemiphytus* Malaise, 1947 and *Fernophytus* Wei, 1997g also have the forewing cells 1R1 and 1Rs combined and the third antennomere shorter than the fourth [88,98]. *Hemocla* Wei, 1995 has the forewing cells 1R1 and 1Rs combined and the third antennomere longer than the fourth [87]. *Heptapotamius* Malaise 1935 has the third antennomere longer than the fourth [31]. *Kattakumia* Zhelokhovtsev, 1964 (*Mongolempria* Ermolenko 1968) has a venation rather similar to that of the new fossil, but with a distinctly shorter forewing cell 2Rs [61].

The new fossil has a clypeus and a wing venation quite similar to those of *Ametastegia* Costa 1882, but its third antennomere is as long as the fourth, while it is distinctly longer in the latter. In addition, the forewing anal crossvein is distinctly less oblique in *Ametastegia* than in the new fossil [30]. *Phrontosoma* MacGillivray 1908 has a clypeus very shallowly emarginated as in the new fossil, but a third antennomere subequal in length to fourth plus fifth antennomeres, and a closed hind wing cell M. *Allantunicus* Smith in Smith & Schiefer, 1997 and *Gulingia* Wei et al., 1997 have a third antennomere longer than fourth [43,115].

Some species of *Empria* Lepeletier & Serville 1828 have a clypeus very similar to that of the new fossil; also, the third antennomere is of the same length as the fourth or nearly so, and the wing venation of the new fossil fits well with that in this genus. The *Empria* spp. have paired opalescent white spots on abdominal tergites 2 to 5 or more, a character that allows an easy attribution of sawflies to this genus [30]. In the case of the new fossil, the presence of a dark, sclerotinized zone on the median part of the tergites together with desclerotinized lateral parts is in accordance with the presence of such opalescent spots.

Thus, we consider that this new fossil can be attributed to the genus *Empria*.

The numerous extant species of *Empria* can be separated using the shape of the claws, head, ovipositor, and colorations, characters hard to determine in the new fossil. Nevertheless, the absence of the hind wing cell M would suggest affinities with the species of the “*candidata*-group” (=subgenus *Parataxonus* comprising *E. candidata* and *E. multicolor*) [27,30,65].

The unique fossil currently listed in the genus *Empria* by Taeger et al. [1] and Prous [27] is *Empria oligocaenica* (Meunier, 1923) from the Oligocene of Rott (Germany) [116]. It was originally attributed to the genus *Dolerus* under the name of *Dolerus oligocaenicus*. Statz [117] excluded this fossil from *Dolerus* and attributed it to *Leucempria* Enslin, 1913, a subgenus of *Empria* after Ross [118], on the basis of wing venation characters only, which are not sufficient to exclude several other empriine genera, while nothing is known on the antenna and the clypeus. The current location of the type specimen is unknown to us. The original description of Meunier [116] does not give any character that could be diagnostic of *Empria ss*. The original photograph of Meunier does not allow to give a precise attribution, except for the possible presence of white spots on the abdominal tergites, suggesting affinities with *Empria*. Statz [117] gave a reconstruction drawing but it is unclear if he has revised or see the type specimen. Therefore, we treat it as an *incertae sedis* within Allantinae *sensu lato*.

## 4. Conclusions

Taeger et al. [1] listed in their catalog of the Symphyta seven species of *Allantus ss*, 33 species of *Emphytus*, *plus* five species not assigned to a subgenus. The extant species are separated based on body characters such as the color of labrum, palps, and head, shape of ovipositor, etc. [111,119,120,121]. As these structures are not preserved in the new fossil, it is not possible to compare it with all the extant species. Nevertheless, as it is the first known fossil of this genus, we prefer to name it rather than leaving the specimen in an open nomenclature. Additionally, the age of the specimen (Miocene) is potentially a sufficient justification to prevent affinities with any extant species of the genus. In addition, its forewing vein 3A reaching posterior wing margin seems to be infrequent among the extant species and could constitute an important difference. 

The attribution of the fossil *Empria* to a species different from the extant one is more complicated to justify because of its younger age; nevertheless, we prefer to do so for the reason stated above.

Niu et al. [2] dated the separation between *Allantus* and *Hemathlophorus* to the middle Eocene–middle Oligocene. Therefore, it is surprising that no older *Allantus* or *Emphytus* fossil has been describe before our study; thus, it is necessary to further explore the tenthredinid fossil record. In fact, numerous specimens are known from fossil imprints or compressions but the fastidious comparisons and sometimes the uncertainties for their placement prevent paleoentomologists to begin their description.

Extant representatives of the genera *Allantus* and *Emphytus* are distributed in the Palearctic, Nearctic, and Oriental regions. *Empria* is also known from the Palearctic, Nearctic, and Oriental regions, but two species are Neotropical. Both genera are known to live on angiosperms (Rosaceae, Salicaceae, Betulaceae, Fagaceae, Polygonaceae, etc.) [111,119,122,123]. Fossils of these families are known in the paleoflora of la Montagne d’Andance and Sainte-Reine [8,13], supporting the putative phytophagy on such angiosperms for *Emphytus miocenicus* sp. nov. and *Empria sammuti* sp. Nov. since the Miocene period.

## References

[1] Taeger A., Blank S.M., Liston A.D. (2010). World catalog of Symphyta (Hymenoptera). Zootaxa.

[2] Nyman T., Onstein R.E., Silvestro D., Wutke S., Taeger A., Wahlberg N., Blank S.M., Malm T. (2019). The early wasp plucks the flower: Disparate extant diversity of sawfly super-families (Hymenoptera: “Symphyta”) may reflect asynchronous switching to angiosperm hosts. Biol. J. Linn. Soc..

[3] Niu G.-Y., Jiang S.-J., Dogan Ö., Korkmaz E.M., Budak M., Wu D., Wei M.-C. (2021). Mitochondrial phylogenomics of Tenthredinidae (Hymenoptera: Tenthredinoidea) supports the monophyly of Megabelesesinae as a subfamily. Insects.

[4] Vilhelmsen L., Engel M.S. (2012). *Sambia succinica*, a crown group tenthredinid from Eocene Baltic amber (Hymenoptera: Tenthredinidae). Insect Syst. Evol..

[5] Demarcq G., Mein P., Ballesio R., Romaggi J.-R. (1989). Le gisement d’Andance (Coiron, Ardèche, France) dans le Miocène supérieur de la vallée du Rhône: Un essai de corrélations marin-continental. Bull. Soc. Géol. Fr..

[6] Brice D. (1965). Recherche sur la flore mio-pliocène de la Montagne d’Andance (Coiron, Ardeche). Ann. Soc. Géol. Nord.

[7] Ehrlich A. (1966). Contribution à l’étude des gisements volcano-lacustres à diatomées de la région de Rochessauve et de St Bauzile. Bull. Soc. Géol. Fr..

[8] Grangeon P. (1958). Contribution à l’étude de la paléontologie végétale du Massif du Coiron (Ardèche) (Sud-Est du Massif Central français). Mém. Soc. D’histoire Nat. D’auvergne.

[9] Riou B. (1988). Les Insectes du Miocène de la Montagne d’Andance (Ardèche). Master Thesis.

[10] Serieyssol K., Gasse F. (1991). Diatomées néogènes du Massif Central français: Quelques faits biostratigraphiques. C.-R. Des Séances De L’académie Des Sci. Paris.

[11] Pastre J.-F., Singer B.S., Guillou H., Pupin J.-P., Riou B. (2004). Chronostratigraphy of the key Upper Miocene (Lower Turolian) sequence of la Montagne d’Andance (Ardèche, France). Implications of new 40Ar/39Ar laser fusion and unspiked K-Ar dating of trachytic tephra and basalts. Bull. Soc. Géol. Fr..

[12] Rey R. (1975). Premières données radiométriques relatives à l’âge du niveau pollinique de Reuver. C.-R. L’Acad. Des Sci. Paris.

[13] Roiron P. (1991). La macroflore d’âge Miocène supérieur des diatomites de Murat (Cantal, France). Implications paléoclimatiques. Palaeontographica.

[14] Wei M.-C., Nie H.-Y. (1998). Generic list of Tenthredinoidea s. str. (Hymenoptera) in new systematic arrangement with synonyms and distribution data. J. Cent. South For. Univ..

[15] Goulet H., Huber J.T. (1993). Hymenoptera of the World: An Identification Guide to Families.

[16] Goulet H. (1992). The genera and subgenera of the sawflies of Canada and Alaska (Hymenoptera: Symphyta). Insects Arachn. Can. Handb. Ser..

[17] Haris A. (2006). New sawflies (Hymenoptera: Symphyta: Tenthredinidae) from Indonesia, Papua New Guinea, Malaysia and Vietnam, with keys to genera and species. Zool. Meded..

[18] Rohwer S.A., Middleton W. (1932). Descriptions of five new Nearctic sawflies of the tribe Hemichroini. Proc. Entomol. Soc. Wash..

[19] Smith D.R. (2003). A synopsis of the sawflies (Hymenoptera: Symphyta) of America South of the United States: Tenthredinidae (Nematinae, Heterarthrinae, Tenthredininae). Trans. Am. Entomol. Soc..

[20] Lacourt J. (2003). Réflexions sur la classification des Blennocampinae, avec description d’un nouveau genre et d’une nouvelle espèce du sud de la France et de Corse (Hymenoptera: Tenthredinidae). Bull. Soc. Entomol. Fr..

[21] Smith D.R. (1972). The South American sawfly genus *Acidiophora* Konow (Hymenoptera: Tenthredinidae). Proc. Entomol. Soc. Wash..

[22] Lacourt J. (1996). Contribution à une révision mondiale de la sous-famille des Tenthredininae. Ann. Soc. Entomol. Fr..

[23] Nie H.-Y., Wei M.-C. (2004). Taxonomic study on the genus *Adamas* Malaise (Hymenoptera: Tenthredinidae). Entomotaxonomia.

[24] Park B., Lee J.-W. (2018). First records of the monotypic genus *Harpiphorus* Hartig (Hymenoptera: Symphyta: Tenthredinidae: Allantinae) from the Eastern Palaearctic region and northern Africa. J. Asia-Pac. Biodivers..

[25] Wei M.-C. (1997). A new subfamily and two new genera of Tenthredinidae (Hymenoptera: Tenthredinomorpha). Entomotaxonomia.

[26] Wei M.-C. (1998). Two new genera of Allantinae from China (Tenthredinidae). Zool. Res..

[27] Prous M. (2012). Taxonomy and phylogeny of the sawfly genus *Empria* (Hymenoptera, Tenthredinidae). Diss. Biol. Univ. Tartu..

[28] Xiao G.-R. (1994). Redescription of the genus *Cladiucha* (Hymenoptera: Tenthredinidae) and descriptions of two new species from China. J. Beijing For. Univ..

[29] Nie H.-Y., Wei M.-C. (2004). On the sawfly genus *Indostegia* Malaise and descriptions of four new species (Hymenoptera, Tenthredinidae, Allantinae). Acta Zootaxonomica Sin..

[30] Smith D.R. (1979). Nearctic sawflies, IV. Allantinae: Adults and larvae (Hymenoptera: Tenthredinidae). United States Agric. Tech. Bull..

[31] Malaise R. (1935). New genera of Tenthredinoidea and their genotypes (Hymen.). Entomol. Tidskr..

[32] Malaise R. (1957). Some Neotropical and Oriental Tenthredinoidea. Entomol. Tidskr..

[33] Malaise R. (1961). New Oriental sawflies (Tenthredinidae). Entomol. Tidskr..

[34] Togashi I., Kondo T. (1979). A new species of the genus *Hemibeleses* Takeuchi (Hymenoptera Tenthredinidae) from Japan. Kontyû.

[35] Xiao G.-r. (1987). A new genus of Tenthredinidae from China (Hymenoptera: Symphyta). Sci. Silvae Sin..

[36] Saini M.S., Vasu V. (1995). A revision of the genus *Ungulia* Malaise (Hymenoptera: Symphyta, Tenthredinidae: Allantinae). J. Bombay Nat. Hist. Soc..

[37] Saini M.S., Vasu V. (1995). Five new species and a revised key to genus *Hemibeleses* Takeuchi from India (Insecta: Hymenoptera: Tenthredinidae: Allantinae). Entomol. Abh..

[38] Saini M.S., Vasu V. (1998). A new genus of Allantinae from India (Hymenoptera: Symphyta: Tenthredinidae). J. Bombay Nat. Hist. Soc..

[39] Saini M.S., Vasu V. (2001). A new genus of Allantinae from India (Hymenoptera: Symphyta: Tenthredinidae). Zoos’ Print J..

[40] Wei M.-C. (1997). A new genus and two new species of Belesesinae (Hymenoptera: Tenthredinomorpha: Blennocampidae) from China. Entomotaxonomia.

[41] Wei M.-C., Yang X.-C. (1997). Hymenoptera: Tenthredinidae (II). Insects of the Three Gorge Reservoir Area of Yangtze River.

[42] Wei M.-C. (2010). Revision of *Megabeleses* Takeuchi (Hymenoptera, Tenthredinidae) with description of two new species from China. Zootaxa.

[43] Wei M.-C., Ouyang G.-M., Huang W.-H. (1997). A new genus and eight new species of Tenthredinidae (Hymenoptera) from Jiangxi. Entomotaxonomia.

[44] Koch F. (1998). The Symphyta of the Ethiopian region. Genus *Neacidiophora* Enslin, 1911 (Insecta: Hymenoptera: Tenthredinidae: Allantinae). Entomol. Abh..

[45] Koch F. (1998). Die Symphyta der Äthiopischen Region. Neue Gattungen und Arten aus der Verwandtschaft der *Neacidiophora*- und *Netroceros*-Gruppe (Hymenoptera: Symphyta: Tenthredinidae: Allantinae). Entomol. Probl..

[46] Wei M.-C., Nie H.-Y. (1997). A new genus and a new species of Belesesini (Hymenoptera: Blennocampidae). J. Cent. South For. Univ..

[47] Wei M.-C., Nie H.-Y. (1998). Three new sawfly genera and species from Hainan Province (Hymenoptera: Tenthredinoidea). J. Cent. South For. Univ..

[48] Wei M.-C., Nie H.-Y., Wu H. (1998). Hymenoptera: Pamphiliidae, Cimbicidae, Argidae, Diprionidae, Tenthredinidae and Cephidae. Insects of Longwangshan Nature Reserve.

[49] Wei M.-C., Nie H.-Y. (1999). A new genus and seven new species of Allantinae (Hymenoptera: Tenthredinidae) from China. J. Cent. South For. Univ..

[50] Saini M.S., Deep J.S. (1999). A review of the Asian genus *Tala* Malaise (Hymenoptera: Tenthredinidae) with description of a new species. Zoos’ Print J..

[51] Saini M.S., Smith D.R., Vasu V. (2002). The sawfly genus *Kambaitina* Malaise (Hymenoptera: Tenthredinidae) in India. Proc. Entomol. Soc. Wash..

[52] Wei M.-C., Niu G.-Y. (2010). Review of the genus *Eriocampopsis* Takeuchi (Hymenoptera: Tenthredinidae) with description of a new species from China. Proc. Entomol. Soc. Wash..

[53] Haris A. (2012). Sawflies from China and Indonesia (Hymenoptera: Tenthredinindae). Nat. Som..

[54] Haris A. (2015). Sawflies from China (Hymenoptera: Tenthredinidae). Nat. Som. Kvár..

[55] Park B., Lee J.-W. (2021). Korean species of the sawfly genus *Hemibeleses* (Hymenoptera: Tenthredinidae), with descriptions of two new species. J. Asia-Pac. Biodivers..

[56] Malaise R. (1934). On some sawflies (Tenthredinidae) from the Indian Museum. Rec. Indian Mus..

[57] Saini M.S., Vasu V. (1996). Taxonomic records on the genus *Darjilingia* Malaise (Hymenoptera: Symphyta: Tenthredinidae: Allantinae). Raffles Bull. Zool..

[58] Konow F.W. (1901). Die Gattung *Sunoxa* Cam. (Hym.). Z. Syst. Hymenopterol. Dipterol..

[59] Smith D.R., Saini M.S. (2003). Review of the Southeastern Asian sawfly genus *Eusunoxa* Enslin (Hymenoptera: Tenthredinidae). J. Hymenopt. Res..

[60] Smith D.R. (2014). A new genus and species of Allantinae (Hymenoptera: Tenthredinidae) from Brazil. Proc. Entomol. Soc. Wash..

[61] Zhelokhovtsev A.N. (1964). A new genus and species of sawflies (Hymenoptera, Tenthredinidae) inhabiting sandy deserts of Middle Asia. Entomol. Obozr..

[62] Ermolenko V.M. (1968). New desert genus and species of the family Tenthredinidae from the Mongolian People’s Republic. Vestn. Zool..

[63] Zhelokhovtsev A.N., Zinov’ev A.G. (1996). A list of the sawflies and horntails (Hymenoptera, Symphyta) of the fauna of Russia and adjacent territories. II. Entomol. Obozr..

[64] Smith D.R., Pratt P.D., Makinson J. (2014). Studies on the Asian sawflies of *Formosempria* Takeuchi (Hymenoptera, Tenthredinidae), with notes on the suitability of *F. varipes* Takeuchi as a biological control agent for skunk vine, *Paederia foetida* L. (Rubiaceae) in Florida. J. Hymenopt. Res..

[65] Prous M., Heidemaa M., Shinohara A., Soon V. (2011). Review of the sawfly genus *Empria* (Hymenoptera, Tenthredinidae) in Japan. ZooKeys.

[66] Benson R.B. (1962). A revision of the Athaliini (Hymenoptera: Tenthredinidae). Bulletin of the British Museum, (Natural History), Entomology.

[67] Benson R.B. (1959). Tribes of the Tenthredinidae and a new European genus (Hymenoptera: Tenthredinidae). Proc. R. Entomol. Soc..

[68] Wei M.-C., Niu G.-Y. (2001). Revision of *Emphytopsis* Wei & Nie (Hymenoptera: Tenthredinidae) with descriptions of seven new species from China and Japan. Zootaxa.

[69] Togashi I. (1992). Japanese sawflies of the genus *Taxonus* Hartig (Hymenoptera; Tenthredinidae) (part 1). Proc. Jpn. Soc. Syst. Zool..

[70] Malaise R. (1949). The genera *Waldheimia*, *Probleta*, and other Neotropical Tenthredinoidea (Hymenoptera). Ark. För Zool..

[71] Wei M.-C., Nie H.-Y. (1999). New genera of Allantinae (Hymenoptera: Tenthredinidae) from China with a key to known genera of Allantini. J. Cent. South For. Univ..

[72] Wei M.-C., Nie H.-Y. (2005). A new genus of Allantini from China (Hymenoptera: Tenthredinidae, Allantinae). Acta Zootaxonomica Sin..

[73] Koch F. (1996). Taxonomie, Phylogenie und Verbreitungsgeschichte der Tribus Xenapateini (Hymenoptera: Tenthredinidae: Allantinae). Entomol. Abh..

[74] Koch F. (1995). Die Symphyta der Äthiopischen Region. 1. Gattung: *Xenapates* Kirby, 1882 (Hymenoptera, Tenthredinidae, Allantinae). Dtsch. Entomol. Z..

[75] Koch F. (1996). Die Symphyta der Äthiopischen Region. Die Gattung *Neoxenapates* Eorsius, 1934 (Hymenoptera: Tenthredinidae: Allantinae). Dtsch. Entomol. Z..

[76] Saini M.S., Vasu V. (2004). The sawfly genus *Allantidea* Rohwer (Hymenoptera: Allantinae) in India. Zoos’ Print J..

[77] Wei M.-C. (2006). A taxonomic study on the genus *Xenapatidea* Malaise (Hymenoptera: Tenthredinidae) from China. Acta Entomol. Sin..

[78] Wei M.-C. (1997). Studies on the tribe Allantini (Hymenoptera: Tenthredinidae)—New taxa and records of Allantini from China. Entomol. Sin..

[79] Wei M.-C., Nie H.-Y. (1999). A new genus and three new species of Allantinae (Hymenoptera: Tenthredinidae) from China. J. Cent. South For. Univ..

[80] Wei M.-C. (2004). A new sawfly genus and species of Allantini (s. str.) (Hymenoptera: Tenthredinidae) with a key to known genera of the tribe. Entomotaxonomia.

[81] Saini M.S., Vasu V. (1996). A new genus and a new species of Allantinae from India (Hymenoptera: Tenthredinidae). Entomol. Ber..

[82] Saini M.S., Deep J.S. (1992). Revision of genus *Monostegidia* Rohwer (Hymenoptera, Tenthredinidae, Allantinae). Dtsch. Entomol. Z..

[83] Togashi I. (1988). Symphyta from Thailand. Steenstrupia.

[84] Saini M.S., Deep J.S. (1992). Revision of genus *Empronus* Malaise from the World (Insecta, Hymenoptera, Tenthredinidae: Allantinae). Entomol. Abh..

[85] Saini M.S., Deep J.S. (1993). Revision of genus *Malachiella* Malaise from the world (Insecta, Hymenoptera, Tenthredinidae: Allantinae). Entomol. Abh..

[86] Saini M.S., Deep J.S. (1992). *Hemkuntus* gen. n. of Allantinae (Hymenoptera: Tenthredinidae) based on a new species from India. Entomon.

[87] Wei M.-C., Wu H. (1995). Hymenoptera: Argidae and Tenthredinidae. Insects of Baishanzu Mountain, Eastern China.

[88] Malaise R. (1947). Entomological results from the Swedish expedition 1934 to Burma and British India, Collected by René Malaise. The Tenthredinoidea of South Eastern Asia. Part III. The *Emphytus*-*Athlophorus* group (Hymenoptera: Tenthredinoidea). Ark. För Zool..

[89] Wei M.-C. (1997). New genera and new species of sawflies from Southwestern China. Zool. Res..

[90] Wei M.-C. (1997). Two new genera of Empriini from China (Hymenoptera: Tenthredinidae). J. Cent. South For. Univ..

[91] Haris A. (2010). Sawflies (Hymenoptera: Tenthredinidae) from South Vietnam. Nat. Som..

[92] Sundukov Y.N. (2010). A new species of the genus *Apethymus* Benson, 1939 (Hymenoptera, Tenthredinidae) from Sikhote-Alin Mountains, Russian Far East. Far East. Entomol..

[93] Togashi I. (2005). Description of a new species of the genus *Apethymus* Benson (Hymenoptera: Tenthredinidae) feeding on *Quercus acutissima* Carruthers (Fagaceae) in Japan. Proc. Entomol. Soc. Wash..

[94] Saini M.S., Singh M., Singh D., Singh T. (1986). Four new species, two each of *Athlophorus* and *Macrophya* (Hymenoptera: Tenthredinidae) from India. J. N. Y. Entomol. Soc..

[95] Saini M.S., Vasu V. (1997). Revision of the genus *Athlophorus* Burmeister from India (Hymenoptera: Symphyta: Tenthredinidae: Allantinae). Isr. J. Entomol..

[96] Saini M.S., Ahmad M. (2012). Four new species of the genus *Athlophorus* Burmeister, 1847 from the Indian Himalayas (Hymenoptera: Symphyta: Tenthredinidae: Allantinae) with a key to Indian species. Acta Zool. Acad. Sci. Hung..

[97] Saini M.S., Vasu V. (1996). Review of the monotypic genus *Hemathlophorus* Malaise from India (Insecta: Hymenoptera: Tenthredinidae: Allantinae). Reichenbachia.

[98] Wei M.-C. (1997). Descriptions of two new genera of Allantinae from China (Hymenoptera: Tenthredinidae). J. Cent. South For. Univ..

[99] Koch F. (1988). Eine neue Allantinengattung und eine neue Art von Taiwan (Hymenoptera, Symphyta, Tenthredinidae). Nachr. Bayer. Entomol..

[100] Wei M.-C., Niu G.-Y. (2008). On the genus *Yushengliua* (Hymenoptera, Tenthredinidae) with description of a new species from China. Acta Zootaxonomica Sin..

[101] Nie H.-Y., Wei M.-C. (1998). A new genus and new species of Belesesinae (Hymenoptera: Blennocampidae). J. Cent. South For. Univ..

[102] Koch F. (1988). Die palaearktischen Arten der Gattung *Apethymus* Benson, 1939 (Hymenoptera, Symphyta, Allantinae). Mitt. Der Münch. Entomol. Ges..

[103] Will H.C. (1933). Wing-venation variations in the rose sawfly, *Emphytus cinctipes* Norton (Tenthredinidae: Hymenoptera). Proc. Pa. Acad. Sci..

[104] Lacourt J. (1989). Description d’un genre nouveau: *Maghrebiella* n. gen., d’Algérie et de Tunisie et redéfinition de deux genres affins: *Allantus* Panzen et *Emphytrus* Klug (Tenthredinidae). Bull. Soc. Entomol. Fr..

[105] Park B., Lee J.-W. (2019). Review of the egg overwintering sawfly genus *Apethymus* Benson (Hymenoptera: Tenthredinidae: Allantinae) from South Korea. J. Asia-Pac. Entomol..

[106] Meunier F. (1914). Nouvelles recherches sur quelques insectes du Sannoisien d’Aix-en-Provence. Bull. Soc. Géol. Fr..

[107] Brues C.T. (1908). New phytophagous Hymenoptera from the Tertiary of Florissant, Colorado. Bull. Mus. Comp. Zool..

[108] Zhang J.-F. (1985). New data of the Mesozoic fossil insects from Laiyang in Shandong. Geol. Shandong.

[109] Abe M., Smith D.R. (1991). The genus-group names of Symphyta and their type species. Esakia.

[110] Ronquist F., Klopfstein S., Vilhelmsen L., Schulmeister S., Murray D.L., Rasnitsyn A.P. (2012). A total-evidence approach to dating with fossils, applied to the early radiation of the Hymenoptera. Syst. Biol..

[111] Magis N. (1999). Les Allantini de la Belgique et des régions limitrophes (Tenthredinidae: Allantinae). Belg. J. Entomol..

[112] Pesarini F. (2001). Contributo alla conoscenza dci Sinfiti della regione balcanico-egea (Hymenoptera, Symphyta). Boll. Del Mus. Reg. Di Sci. Nat. Di Torino.

[113] Park B., Smith D.R., Lee J.-W. (2018). Discovery of the genus *Dinax* Konow (Hymenoptera: Symphyta: Tenthredinidae: Allantinae) in Japan and South Korea. J. Asia-Pac. Biodivers..

[114] Rohwer S.A. (1909). Notes on Tenthredinoidea, with descriptions of new species. (Paper III). Can. Entomol..

[115] Smith D.R., Schiefer T.L. (1997). A new genus and species of Allantinae (Tenthredinidae) from Southeastern United States. Proc. Entomol. Soc. Wash..

[116] Meunier F. (1923). Sur quelques insectes de l’Aquitanien de Rott. Misc. Entomol..

[117] Statz G. (1936). Über alte und neue fossile Hymenopterenfunde aus den tertiären Äblagerungen von Rott am Siebengebirge. Dechen. Verh. Des Nat. Ver. Der Rheinl. Westfal..

[118] Ross H.H. (1937). A generic classification of the Nearctic sawflies (Hymenoptera, Symphyta). III. Biol. Monogr..

[119] Berland L. (1947). Hymenoptera Tenthredoides. Faune De Fr..

[120] Benson R.B. (1952). Hymenoptera. 2. Symphyta, section (b). Handb. Identif. Br. Insects.

[121] Wong H.R. (1966). A new species of *Allantus* Panzer on Birch (Hymenoptera: Tenthredinidae). Can. Entomol..

[122] Okutani T. (1967). Food-plants of Japanese Symphyta (II). Jpn. J. Appl. Entomol. Zool..

[123] Park B., Lee J.-W. (2019). Six new records of Allantinae (Hymenoptera: Symphyta: Tenthredinidae) from Korea, with a revised key to the allantine genera. J. Asia-Pac. Biodivers..

